# Global Proteomic Analysis of Colorectal Cancers Stratified by Microsatellite Instability Subtype Reveals Protein Differences

**DOI:** 10.64898/2026.01.28.702312

**Published:** 2026-01-29

**Authors:** Fernando Tobias, Emily R. Sekera, Xingzhao Xiong, Fei Fang, Heather Hampel, Rachel Pearlman, Xiaowen Liu, Liangliang Sun, Amanda B. Hummon

**Affiliations:** 1: Department of Chemistry and Biochemistry and the Comprehensive Cancer Center, The Ohio State University, Columbus, Ohio, 43210, USA.; 2: Deming Department of Medicine, School of Medicine, Tulane University, New Orleans, LA 70112, USA.; 3: Department of Chemistry, Michigan State University, East Lansing, MI 48824, USA.; 4: City of Hope, Duarte Cancer Center, Duarte, CA 91010, USA.; 5: Department of Internal Medicine and the Comprehensive Cancer Center, The Ohio State University, Columbus, Ohio, 43210, USA.

**Keywords:** Lynch Syndrome, Proteomics, Inflammatory Markers, MSH-H, MLH1 hypermethylation, Double Somatic MMR Mutation

## Abstract

Lynch syndrome, historically known as hereditary nonpolyposis colorectal cancer, is caused by germline mutations in the DNA mismatch repair (MMR) genes, *MLH1, MSH2 (EPCAM), MSH6*, and *PMS2*. While the genetic changes associated with Lynch Syndrome have previously been characterized, there have not been studies of the associated proteomic alterations, in part because of the limited availability of primary samples and the absence of *in vitro* model systems. In this study, the first large-scale tissue proteomic assessment of Lynch Syndrome samples as well as three other subtypes of colorectal cancer was completed with specimens from the Ohio Colorectal Cancer Prevention Initiative. The cohort contained three groups of microsatellite unstable (MSI-high) CRC patients (Lynch syndrome, double somatic MMR mutation, and MLH1 hypermethylation) and a group of microsatellite stable (MSS) CRC patients. A total of 122 tumor and complimentary normal mucosa samples from 61 patients were evaluated using label-free bottom-up proteomic analysis. Hierarchical clustering analysis of the global proteome showed that the MSS group was significantly different than the three MSI-high groups. Of the 1,084 proteins found to be dysregulated across all four colorectal cancer subtypes, there were age at diagnosis associated shifts in proteins correlated with tumor proliferation and immune regulation for the Lynch syndrome and Double Somatic samples. The proteins TPD52, GMDS, and DSP showed increased protein abundance correlated with older age at diagnosis. In addition, the Lynch syndrome samples showed substantial sex-based differences in immune and inflammatory pathways, for example, downregulation of ZG16, DIS3, and WDR43. This study fills a critical gap as the first proteomic characterization of Lynch syndrome samples to date. Data are available via ProteomeXchange with identifier PXD073693.

## Teaser

This is the first study of the global proteomic differences between Lynch Syndrome and other forms of colorectal cancer.

## Introduction:

Colorectal cancer (CRC) is the third most common cancer diagnosed worldwide in both men and women.^1^ The majority of CRC cases are microsatellite stable (MSS), with 15% exhibiting high microsatellite instability (MSI-H). MSI is a condition characterized by the accumulation of errors in microsatellite repeat sequences due to a deficient DNA mismatch repair (MMR) system. This deficiency can arise from three primary mechanisms: Lynch syndrome (LS), double somatic MMR gene mutations, and epigenetic silencing of *MLH1* (hypermethylation).

- Lynch syndrome (LS) is the most common form of hereditary CRC and causes a genetic predisposition to other cancer types, including endometrial, ovarian, stomach, and urothelial, among others. Diagnosing LS is extremely useful for treatment implications of a current cancer as well as future cancer prevention for an individual and their family members.
- Tumors caused by “double somatic” MMR mutations are a more recently described phenomenon that involves two separate somatic mutations in the same MMR gene on opposite alleles (*in trans*) within the same cell, leading to MSI in the absence of a germline mutation and typically presenting in a sporadic manner.^2, 3^
- The most common cause of MSI-H CRC is epigenetic silencing (ES) due to hypermethylation of the *MLH1* promoter which inhibits expression of the MLH1 gene without altering the DNA sequence.^4^ *MLH1* promoter hypermethylation is typically acquired and only rarely inherited. MSI-H tumors typically have a better prognosis and are more likely to respond to immunotherapies, such as immune checkpoint inhibitors.
- MSS colorectal cancers, characterized by stable microsatellites, harbor an intact MMR system. These tumors tend to follow a different tumorigenesis pathway, marked by chromosomal instability and often involve somatic mutations in genes such as *KRAS* and *TP53*. MSS tumors generally have a poorer prognosis and are less responsive to immunotherapy compared to MSI tumors.

A better understanding of these four types of CRC at the molecular level could prove pivotal in improving early diagnosis and designing therapeutic options.^5^

**Figure 1:**
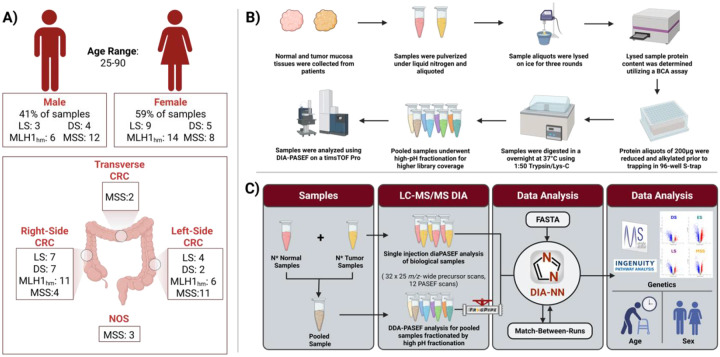
Summary of Samples and Sample Preparation and Analysis. **(A)** Breakdown of the 122 tumor and normal mucosa samples from the 61-patient cohort across the four colorectal cancer (CRC) subtypes: Microsatellite Stable (MSS), MLH1 Hypermethylation (MLH1_hm_), Lynch Syndrome (LS), and Double Somatic (DS). Additional information on patient age at diagnosis, gender and original tumor location are also provided. **(B)** Overview of sample preparation for patient tumor and mucosa samples. **(C)** Overview of DIA analysis from generation of the pooled samples for mucosa and tumors to data analysis.

Much effort has been devoted to understanding CRC, including MSI tumors, at the genome and transcriptome levels. However, these analyses cannot accurately reflect the protein-level information as post-transcriptional regulation can modulate protein expression and post-translational modifications (PTMs) can influence protein functions. The generation of proteome-level information is imperative for bridging the genotype and phenotype of CRC. Mass spectrometry (MS)-based proteomics has become increasingly popular for high-throughput and large-scale measurement of proteins in cancer cells and tissues for pursuing a better understanding of underlying molecular mechanisms of cancer progression and for discovering new protein biomarkers for more accurate and earlier diagnosis.^6–13^ However, there are limited proteomics datasets generated from LS samples, most likely due to the limited availability of clinically obtained tissue. To discover new protein biomarkers, we performed the first large-scale quantitative proteomics study of LS tissue samples and compared the tissue proteome landscape across different subtypes of CRC: MSS, MSI: LS, MSI: Double Somatic (DS), and MSI: *MLH1* hypermethylation (*MLH1*_hm_), as well as healthy controls (Table 1 and Figure 1). By integrating demographic metadata with quantitative proteomics, this study provides critical insights into the molecular heterogeneity of CRC and the influence of patient-specific factors on tumor biology, setting the stage for future biomarker development and precision medicine approaches in CRC.

## RESULTS

The samples were acquired through The Ohio Colorectal Cancer Prevention Initiative.^14^ The CRC samples included in this study are novel due to their geographical specificity and contain a diverse age group. This cohort included rich metadata to better understand the phenotypic and proteomic data shared in this study. We uncovered 1,084 proteins dysregulated across all four CRC subtypes, with subtype-specific expression patterns that highlight potential diagnostic biomarkers. As expected, our findings reveal substantial differences between the proteomes of MSS and MSI-high patients. We also observe age-associated shifts in tumor proliferation and immune regulation, particularly in LS and DS subtypes, and sex-based differences in inflammatory and immune pathways, most notably in LS and DS.

### Global protein expression differences between CRC subtypes

**Figure 2:**
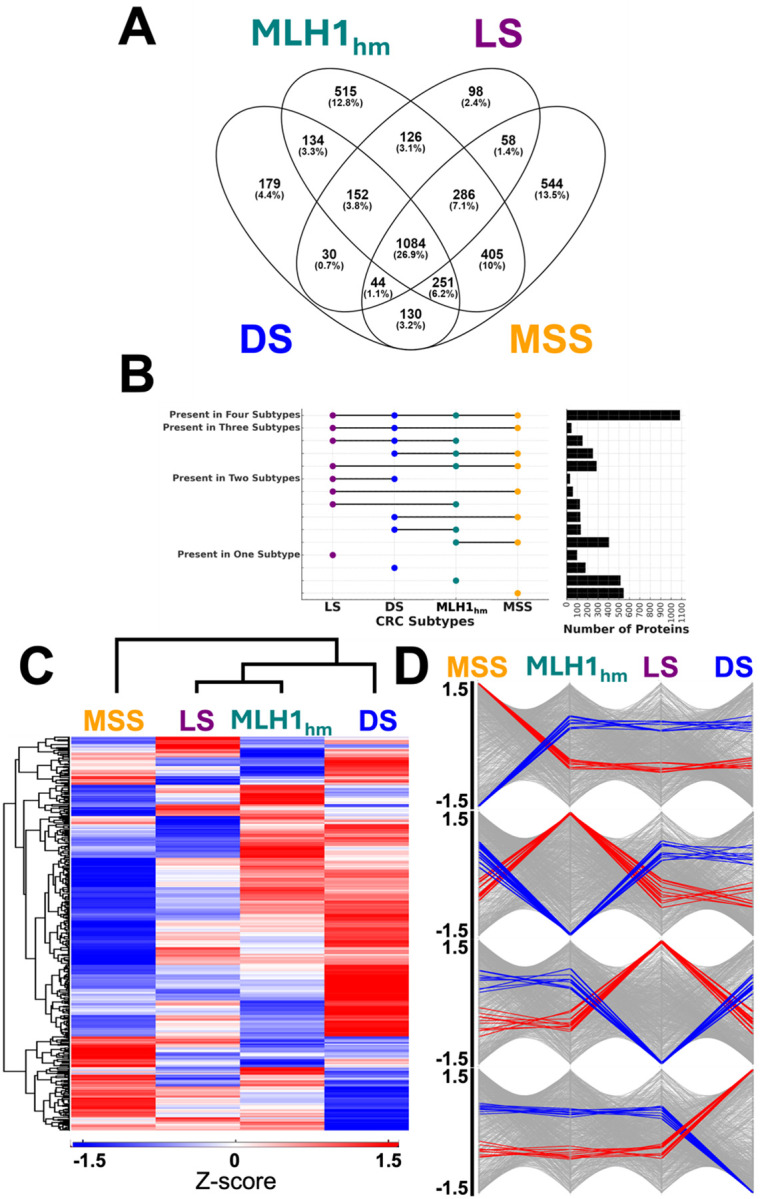
Overlap of Dysregulated Proteins Across CRC Subtypes. **(A)** Venn diagram illustrating the overlap of 4,066 dysregulated proteins across the four colorectal cancer (CRC) subtypes: Microsatellite Stable (MSS), MLH1 Hypermethylation (MLH1_hm_), Lynch Syndrome (LS), and Double Somatic (DS). Each segment of the diagram shows the number and percentage of proteins unique to or shared between subtypes. 1,084 proteins, found at the intersection of all four subtypes, represent the common dysregulated proteins. **(B)** UpSet plot visualizing the overlap of dysregulated proteins across the four CRC subtypes. The left panel shows dots representing protein presence in each subtype, with lines connecting overlapping subtypes. The right panel displays the number of proteins in each overlap group. This visualization complements the Venn diagram by clearly quantifying the extent of protein overlap among the subtypes. **(C)** Hierarchical clustering heatmap of the 1,084 common dysregulated proteins, showing their z-score-normalized expression across the four subtypes. The clustering highlights subtype-specific patterns of protein expression, with red indicating higher abundance and blue indicating lower abundance. **(D)** Abundance trend plots, as normalized by their z-score, of the 1,084 common dysregulated proteins across the four subtypes, emphasizing the top 10 upregulated (red) and top 10 downregulated (blue) proteins specific to each subtype. These plots illustrate how protein dysregulation trends vary between subtypes, reflecting key differences in CRC biology and tumor progression mechanisms.

Analysis of whole proteome expression alterations between tumor and normal tissues revealed 4,066 significantly dysregulated proteins in at least one CRC subtype. Within each subtype, we identified 2,005, 1,879, 2,954, and 2,803 dysregulated proteins for the Double Somatic (DS), Lynch Syndrome (LS), MLH1 hypermethylation (MLH1_hm_), and Microsatellite Stable (MSS) subtypes, respectively. Among these, 179, 98, 515, and 544 proteins, respectively, were uniquely dysregulated in each subtype, while 1,084 dysregulated proteins were common to all subtypes (Figure 2A). The UpSet plot (Figure 2B) provides a quantitative visualization of the overlap between dysregulated proteins across CRC subtypes. The bar graphs illustrate the extent of protein sharing between subtypes, with 1,084 dysregulated proteins being common to all subtypes. To further characterize these shared dysregulated proteins, hierarchical clustering was performed, generating a heatmap that distinctly separates CRC subtypes based on their protein expression profiles (Figure 2C). The detected protein changes in the MSS cohort were significantly differentiated from the three MSI-high patient cohorts (Figure 2C). The branching in the heat map shows MSS as distinct from the three MSI-high variants. Within the three MSI-high cohorts, LS and MLH1_hm_ were more similar, compared to the DS group. A complete list of all dysregulated proteins is available in the Supplemental Information. Supplemental Tables S1–3 provide the Top 10 upregulated and downregulated proteins and Supplemental Figures S1–3 show the Top Biological Processes from Gene Ontology analysis in MSS, MLH1_hm_, and DS subtypes, respectively.

To investigate subtype-specific protein dysregulation, we examined the distribution of the 1,084 shared proteins across the four CRC subtypes to identify proteins with subtype-enriched patterns. Trendlines were generated to depict global expression distributions, while the top 10 upregulated and downregulated proteins specific to each subtype were highlighted (Figure 2D). This analysis revealed distinct proteomic signatures defining each subtype, characterized by proteins exhibiting high Z-scores (upregulated) or low Z-scores (downregulated), thereby providing insights into the molecular mechanisms driving tumor progression. As observed in the unsupervised clustering, the abundance trend lines for MSS showed Z scores that were markedly different than the three MSI-high cohorts, indicating pronounced differences in protein regulation.

The top 10 upregulated and downregulated proteins are listed in Table 2, which lists the normalized Z-scores and protein expression changes as log2-fold differences between tumor and normal mucosa samples across each subtype. The LS subtype showed elevated expression of several RNA-processing and chromatin-associated proteins, including OGFR, RCN1, TGFB1, and WDR8. Notably, TGFB1 has been implicated in colorectal cancer progression through its role in tumor microenvironment modulation and immune response regulation.^15^ Downregulated proteins include ZG16, F13A1, and PTGFRN. ZG16 has been shown to inhibit proliferation, invasion, and migration of CRC cells, and its overexpression suppresses epithelial-mesenchymal transition and Wnt/β-catenin signaling pathways.^16^ Additionally, ZG16 negatively correlates with PD-L1 expression, suggesting a role in modulating immune responses in CRC.^17^ Several proteins (e.g., DIS3, DDX18, WDR43) involved in ribosome biogenesis and RNA surveillance appeared downranked in LS based on Z-scores, despite elevated abundance, suggesting differential pathway engagement across subtypes. Alterations in ribosome biogenesis are a hallmark of CRC, contributing to enhanced protein synthesis and tumor progression.^18^

### Biological Process Enrichment Analysis of Dysregulated Proteins

**Figure 3:**
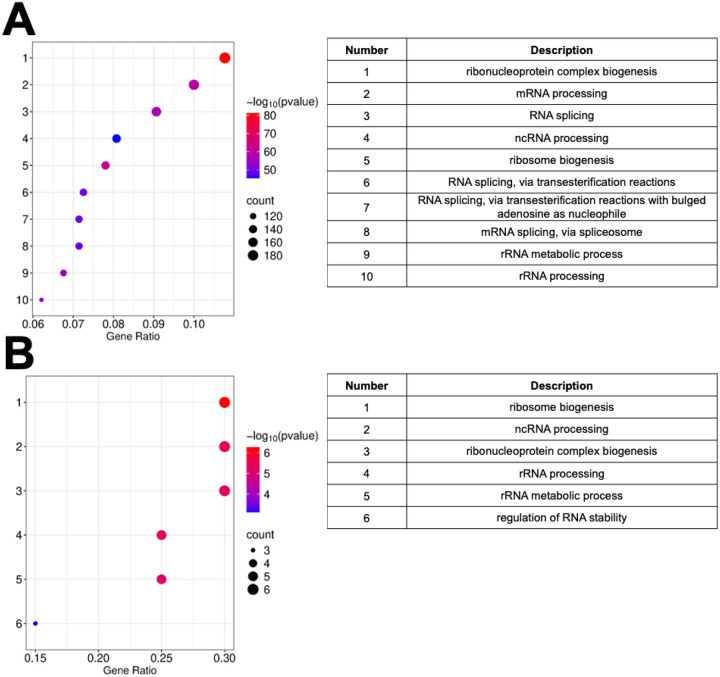
Top Biological Process (BP) terms from Gene Ontology (GO) analysis for the Lynch Syndrome (LS) subtype. The bubble size represents the number of genes associated with each term, while the color indicates statistical significance, expressed as the −log_10_(p-value). **(A)** The GO analysis of the 2,803 dysregulated proteins and **(B)** is the GO analysis based on the top 10 upregulated and downregulated proteins in LS subtype, which are part of the 1,084 shared dysregulated proteins.

For the LS subtype, Figure 3A illustrates the GO enrichment analysis results on all 2,803 significantly dysregulated proteins in LS tumors relative to matched normal mucosa. This overview revealed strong enrichment for RNA- and translation-related biological processes, including ribonucleoprotein complex biogenesis, mRNA processing, RNA splicing, and ribosome biogenesis.^18, 19^ These terms ranked highest by both gene ratio and statistical significance (–log_10_ p-values > 70), indicating a global shift in transcriptional and translational machinery in LS tumors. Additional enriched terms—such as ncRNA processing and spliceosome-mediated transesterification reactions—pointed to widespread perturbation of post-transcriptional regulation, consistent with the elevated expression of splicing and ribosome biogenesis factors identified in LS, such as DIS3, DDX18, and WDR43.^20^ In Figure 3B, a more targeted GO analysis was performed using only the top 10 upregulated and top 10 downregulated proteins in LS (Table 2). These proteins were drawn from a core set of 1,084 shared dysregulated proteins present across all CRC subtypes but ranked highest in LS by Z-score. Despite this narrower focus, the analysis revealed enriched terms that mirrored the broader LS proteomic signature, including mRNA splicing via spliceosome, ncRNA metabolic process, and regulation of translation. Unique to this GO analysis set were GO terms like chromatin silencing at rDNA and cytoplasmic translation, supporting a model in which LS tumors selectively rewire key pathways governing ribosome function, gene silencing, and post-transcriptional control. This alignment between global and top-ranked GO terms suggests that even among commonly dysregulated proteins across CRC, LS tumors exhibit distinct regulatory prioritization reflective of MMR deficiency and heightened genomic stress.^21^

### Age-Associated Protein Dysregulation in CRC

The LS subtype exhibited the strongest age at diagnosis dependent dysregulation, with several proteins displaying high positive correlations with an older age at diagnosis. Among them, TPD52 (0.93), GMDS (0.85), and DSP (0.84) showed the highest correlations, suggesting that tumor proliferation and metabolic activity could become more pronounced with age of diagnosis in LS patients. Conversely, proteins associated with immune function and extracellular matrix (ECM) remodeling, such as MXRA5 (−0.88), AEBP1 (−0.87), and ARG1 (−0.84), were negatively correlated with age at diagnosis, indicating a possible decline in immune regulation and ECM integrity in older LS patients, which may facilitate tumor progression and metastasis. However, it is worth noting that the age at diagnosis is not necessarily the age of the tumor itself, as the tumor may have been present for years prior to diagnosis and staging information was not available to assess whether the correlation is truly with advancing age or stage.

**Figure 4:**
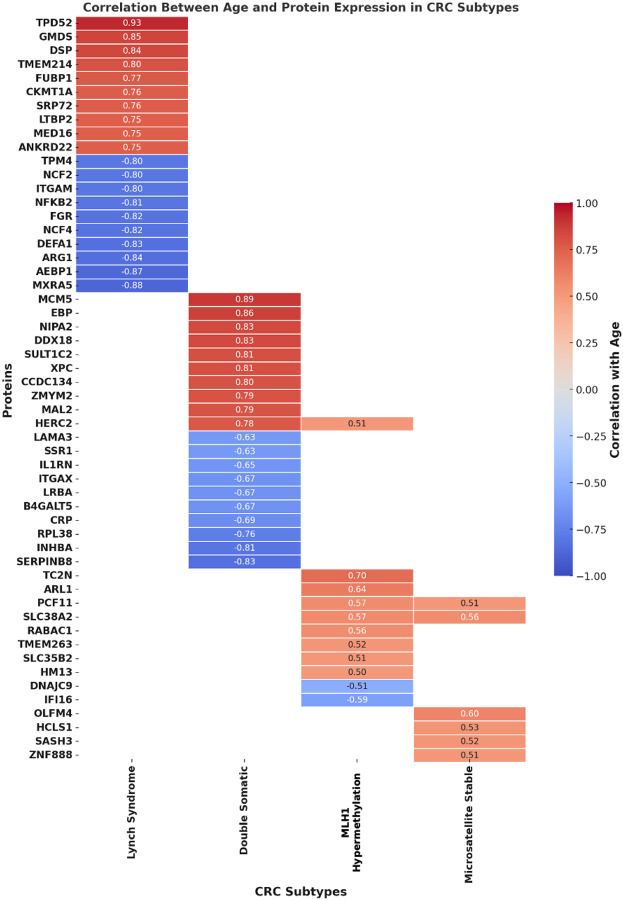
Correlation Between Age at Diagnosis and Protein Expression in CRC Subtypes. This heatmap displays the top 20 proteins most correlated with age at diagnosis in colorectal cancer (CRC) subtypes, highlighting a subset of a larger dataset of dysregulated proteins: Double Somatic (DS) = 196 total, Lynch Syndrome (LS) = 271, MLH1 Hypermethylation (MLH1_hm_), = 11, and Microsatellite Stable (MSS) = 6. A positive correlation (red) indicates proteins that increase in expression with age at diagnosis, whereas a negative correlation (blue) represents proteins that decrease with age at diagnosis. This visualization highlights age-dependent molecular alterations that may contribute to tumor progression, metabolic shifts, and immune regulation across CRC subtypes.

The resulting correlation coefficients, shown in Figure 4, highlight distinct sets of proteins whose expression is strongly associated with age at diagnosis in a subtype-specific manner. Based on these correlations, we selected the top two proteins per subtype—those showing the strongest positive or negative correlation with age—and visualized their age-abundance relationships in Figure S4. This figure presents a combined view of age-dependent protein expression: scatter plots illustrate normalized protein abundance as a function of age at diagnosis, while adjacent boxplots summarize the age distribution of the samples contributing to each protein’s trend. In LS tumors, TPD52 exhibited a strong positive correlation with age (r = 0.93), consistent with increased oncogenic signaling in older patients, while MXRA5 showed a strong negative correlation (r = −0.88), aligning with reduced extracellular matrix remodeling in patients diagnosed with an MMR deficient tumor later in life. In MSS tumors, OLFM4 expression rose with age (r = 0.60), implicating stemness or immune evasion processes, while SLC38A2 showed a modest decrease (r = −0.51), hinting at altered amino acid transport in older MSS-diagnosed patients.

**Figure 5:**
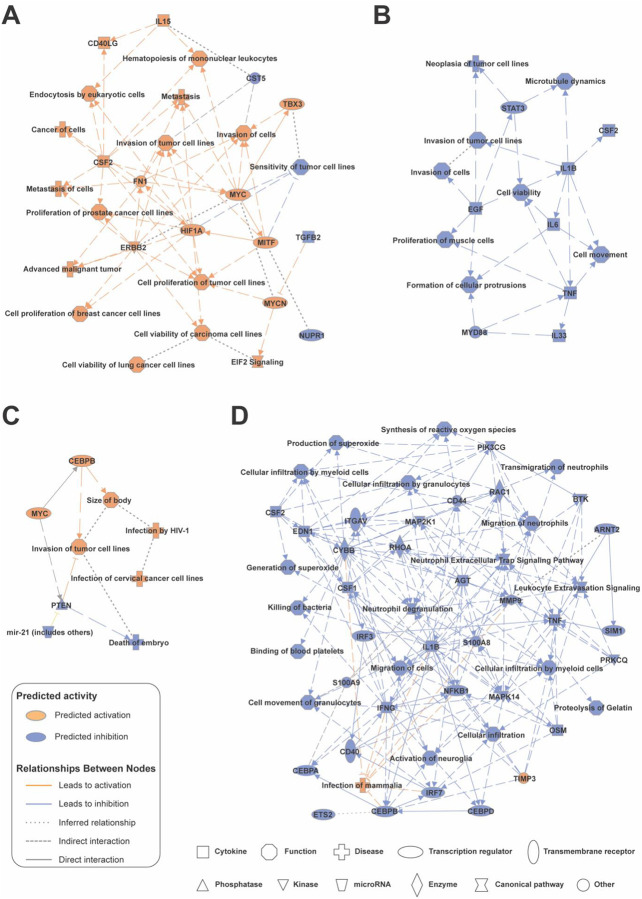
Ingenuity Pathway Analysis (IPA) Graphical Summary of Age-at-Diagnosis-Related Proteomic Changes in Double Somatic (DS) and Lynch Syndrome (LS) Tumors. (A) Positive correlation of DS proteins with age at diagnosis, highlighting pathways and biological processes enriched in age at diagnosis associated upregulated proteins. (B) Negative correlation of DS proteins with age at diagnosis, illustrating pathways and cellular functions associated with age-associated downregulated proteins. (C) Positive correlation of LS proteins with age at diagnosis, revealing key regulatory networks and biological functions linked to increased protein expression with aging. (D) Negative correlation of LS proteins with age at diagnosis, depicting pathways and processes influenced by proteins that decrease in abundance with age at diagnosis.

To further understand the biological implications of age-at-diagnosis associated dysregulation in the subtypes, Ingenuity Pathway Analysis (IPA) Graphical Summaries were generated to explore key pathways and molecular mechanisms (Figure 5). The DS subtype (Figure 5A and B) displayed a distinct age-associated molecular profile. Activated pathways from the proteins that are positively correlated with age included HIF1A signaling, extracellular matrix remodeling via FN1, and MYC-driven proliferation, reflecting enhanced metastatic and hypoxic adaptation in DS patients diagnosed at older ages. In the LS subtype (Figure 5C and D), age-associated dysregulation was characterized by the activation of MYC signaling and CEBPB-mediated transcription, both of which promote tumor proliferation and invasion. Activated functions such as invasion of tumor cell lines and tumor progression underscore the heightened oncogenic potential in aging LS patients. Conversely, suppressed pathways, including PTEN signaling and miR-21 regulation, reflect a loss of tumor-suppressive capacity with age at diagnosis.

### Sex-Associated Protein Dysregulation in CRC

**Figure 6:**
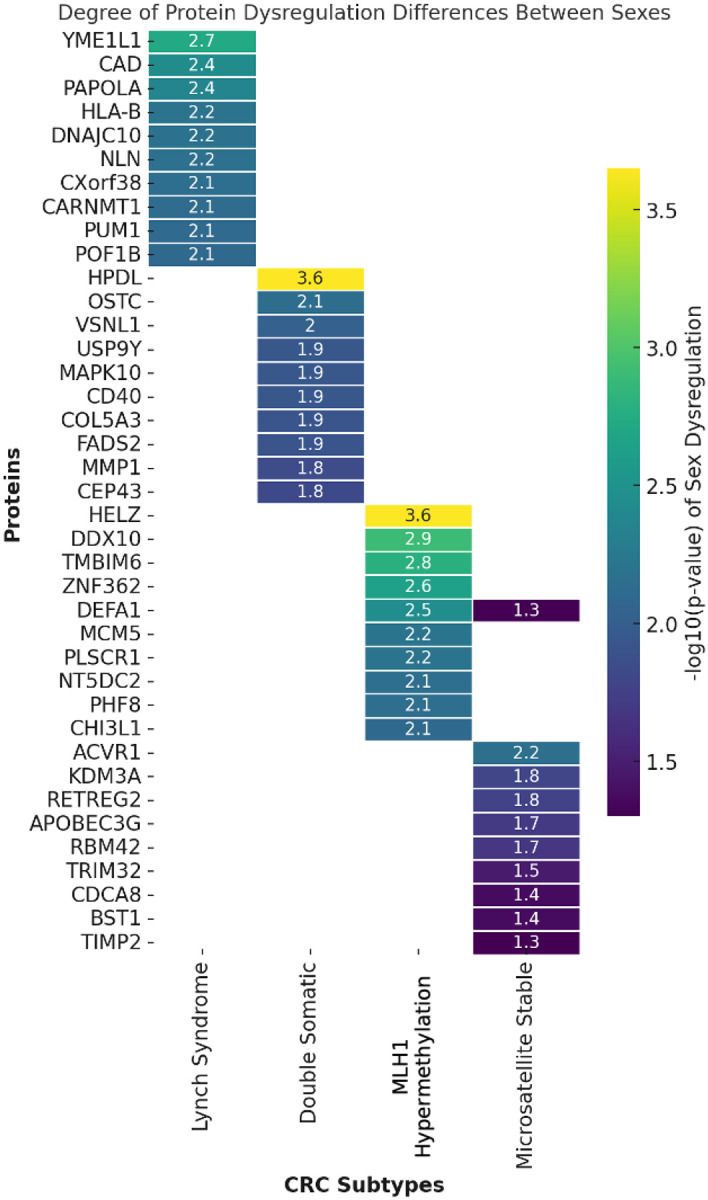
Degree of Protein Dysregulation Differences Between Sexes. This heatmap presents the top proteins most significantly associated with sex-based differences in dysregulation, selected from a larger dataset where DS = 19, LS = 69, MLH1_hm_), = 63, and MSS = 10. The values are represented as −log10(p-values), where higher values (red) indicate more significant sex-dependent differences in protein expression, while lower values (blue) suggest minimal differences between male and female patients. This visualization provides insight into sex-associated variability in protein dysregulation, which may be influenced by hormonal, genetic, or immune-related factors that impact CRC tumor biology and potential therapeutic responses.

We next examined the statistical significance of sex-based differences in protein dysregulation across the CRC subtypes (Figure 6). In LS, proteins like YME1L1 (2.71), CAD (2.43), and PAPOLA (2.37) displayed significant differences, many of which are involved in mitochondrial function and nucleotide metabolism. These findings suggest that sex-related metabolic and immune differences may play an important role in tumor progression in LS and MLH1_hm_ subtypes. Conversely, the DS and MSS subtypes exhibited fewer proteins with significant sex-associated differences. Notable examples in DS included HPDL (-log_10_(p) = 3.65) and OSTC (2.14), while MSS showed only moderate sex-specific dysregulation, with proteins such as ACVR1 (2.15) and KDM3A (1.80). The limited number of proteins in DS and MSS indicates that sex may play a less prominent role in these subtypes.

**Figure 7:**
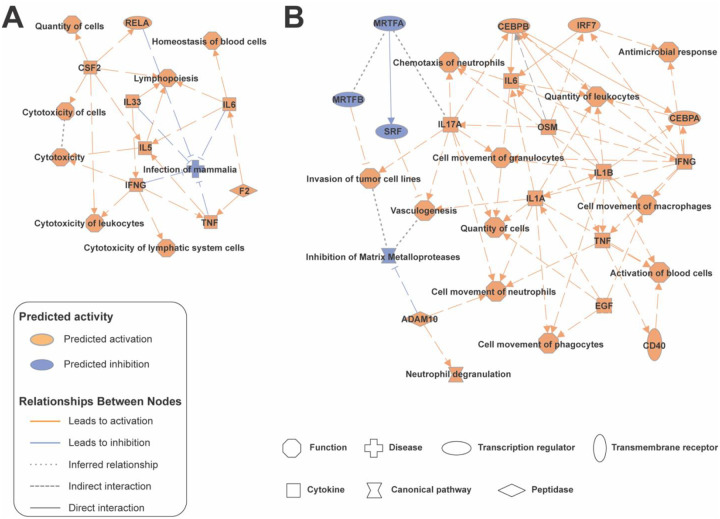
Ingenuity Pathway Analysis (IPA) Graphical Summary of Sex-Related Proteomic Differences in Lynch Syndrome (LS) and MLH1 Hypermethylation (MLH1_hm_) Tumors. (A) IPA graphical summary of LS proteins correlated with sex, highlighting key regulatory networks, pathways, and biological processes that differ between male and female LS tumors. (B) IPA graphical summary of MLH1_hm_ proteins correlated with sex, illustrating major molecular interactions and functional pathways associated with sex-dependent proteomic differences in MLH1_hm_ tumors.

IPA Graphical Summaries for the LS and MLH1_hm_ subtypes (Figure 7) provided insights into the biological processes and pathways influenced by sex-associated dysregulation. In the LS subtype, immune-related processes such as lymphopoiesis, cytotoxicity of leukocytes, and homeostasis of blood cells were predicted to be activated. The activation of transcription factors like RELA (NF-κB p65) suggests heightened inflammatory signaling, while the suppression of pathways like IL33 signaling and infection of mammalian cells highlights a nuanced balance in immune regulation. These findings suggest a strong role for immune modulation in sex-specific tumor biology in LS.

## DISCUSSION

This study provides a comprehensive investigation of protein dysregulation across four CRC subtypes—Lynch Syndrome (LS), Double Somatic (DS), MLH1 Hypermethylation (MLH1_hm_), and Microsatellite Stable (MSS)—with a focus on demographic influences such as age and sex. We highlighted the heterogeneity of CRC and the biological processes driving tumor progression in different subtypes. To date, there have been no large-scale proteomic studies that compare MSI-H CRC samples (some due to genetic predisposition) and MSS CRC samples. While LS has long been characterized by MMR proteins MLH1, MSH2, MSH6, and PMS2, we hypothesized that an unbiased mass spectrometric analysis of primary tissues would yield additional protein biomarkers. Previous large-scale analyses of CRC samples have affirmed that CRCs can be stratified into distinct molecular subgroups, which provides context for the proteomic differences observed among LS, DS, MLH1_hm_, and MSS tumors.^9, 10, 22, 23^ Notably, The Cancer Genome Atlas (TCGA) study^8^ established that about 16% of CRCs are hypermutated – most of these have high MSI with MLH1 promoter hypermethylation (the CIMP-high, sporadic MSI pathway) or, in a minority, biallelic mismatch-repair gene mutations.^8^ This corresponds to the MLH1_hm_, LS, and DS categories in the current study.

### Global Subtype Differences and Shared Proteomic Differences Across CRC

Across the four subtypes, our proteomic analysis identified 4,066 proteins that were significantly dysregulated in tumor tissue relative to normal controls. Notably, 1,084 of these proteins were commonly dysregulated across all subtypes (Figure 2A), suggesting a core set of proteomic alterations underpinning colorectal tumorigenesis regardless of mismatch repair or microsatellite status. Functionally, this shared dysregulated proteome reflects fundamental hallmarks of CRC biology. We observed widespread upregulation of proteins involved in chromatin modification, RNA processing/translation, and DNA replication/repair, consistent with the enhanced proliferative and biosynthetic demands of cancer cells.^24^ This is in line with prior proteomic studies showing elevated abundance of chromatin regulators, gene expression machinery, and DNA repair factors in CRC.^24^ For example, many histone modifiers, RNA helicases, and ribosomal proteins appear in the set of 1,084 dysregulated proteins, indicating that sustained proliferative signaling in CRC is universally supported by boosting transcriptional and translational capacity – a known cancer hallmark.^25–28^ Likewise, numerous metabolic enzymes and stress-response chaperones were commonly upregulated, reflecting the reprogrammed metabolism and stress tolerance characteristic of tumors.

Conversely, proteins associated with normal colonic epithelial differentiation and structural integrity tended to be consistently downregulated in all subtypes. One prominent example is zymogen granule protein 16 (ZG16), a secretory lectin normally abundant in goblet cells. ZG16 was significantly under-expressed in every subtype, in agreement with reports that it is one of the most downregulated proteins in CRC tissues.^29, 30^ Loss of ZG16, which can enhance dendritic cell activation and antitumor immunity, exemplifies how tumors commonly extinguish normal mucosal defense mechanisms as they progress.^29^ We also found uniform suppression of certain extracellular matrix and cell adhesion proteins in the set of 1,084 dysregulated proteins, echoing prior findings that CRC exhibits decreased expression of core extracellular matrix components during the adenoma–carcinoma transition.^24^ Taken together, these shared proteomic alterations highlight the universal activation of oncogenic pathways (e.g. Wnt/β-catenin and Myc programs driving protein synthesis) and the loss of homeostatic functions (barrier, differentiation markers) that define colorectal cancer, consistent with established hallmarks of CRC pathogenesis.^24, 31^

Despite this common core of dysregulation, the expression profiles of these 1,084 shared proteins were not identical across subtypes. Hierarchical clustering and trend analysis (Figure 2C–D) revealed that even within the shared set, subtype-specific expression biases could be discerned. In particular, the proteome of MSS tumors was significantly altered from the MSI-high cohorts (Figure 2C). Each subtype exhibits a distinctive quantitative pattern superimposed on the common dysregulations. This suggests that while all CRCs co-opt similar biological processes, the degree or way these processes are modulated can differ by subtype.

LS tumors showed a proteomic profile indicative of heightened chromatin/RNA processing activity coupled with an immunomodulatory tumor microenvironment. Among the 1,084 shared dysregulated proteins, LS tumors had some of the highest relative upregulation of WDR82, OGFR, and TGFβ1 (TGFB1) as shown in Figure 3. Consistent with this, LS tumors showed relative downregulation of several immune-related and ribosomal biogenesis proteins. Notably, ZG16 was among the most downregulated in LS (even against a low baseline in other subtypes). Given ZG16’s role in promoting mucosal immunity and T-cell mediated tumor suppression,^29^ its further loss in LS tumors reinforces the idea of immune regulatory down-tuning.

### Age-Associated Proteomic Signatures in CRC

Among the CRC subtypes, DS and LS tumors had the highest protein abundance correlations relative to patient age at diagnosis. In contrast, MSS tumors exhibited minimal age-related dysregulation, indicating age-independent tumor progression mechanisms in these subtypes. DS MSI-high tumors often occur in the right colon and tend to be large, locally invasive masses with less nodal metastasis.^32, 33^ Such features suggest a hypoxic tumor microenvironment in older DS cases, which can drive HIF1A (HIF-1α) pathway activation. Indeed, HIF-1α is frequently upregulated in advanced CRC and correlates with angiogenesis, invasion, and poor prognosis.^34^ This supports the claim that tumors in older DS patients may show increased HIF1A signaling. In addition, MYC-driven proliferative programs appear enhanced with age: proteomic profiling of CRC patients revealed that MYC target gene sets were more highly expressed in tumors from older patients.^35^ Similarly, the extracellular matrix protein fibronectin (FN1) – linked to invasion and migration – is elevated in CRC tumors and its expression correlates positively with patient age.^36^ These findings are consistent with DS tumors from older patients exhibiting higher activity of HIF1A, MYC, and FN1-related pathways.

LS patients usually develop CRC at relatively young ages (commonly 40s–50s), making it challenging to find studies stratifying LS tumors by age.^37^ In the present study, the top three highly correlated protein abundance with advancing age at diagnosis were TPD52, GMDS and DSP. Cumulatively, this suggests a shift to more aggressive biological pathways, such as enhanced proliferation (TPD52),^38, 39^ immune evasion and altered adhesion (GMDS),^40^ and abnormal epithelial integrity or compensatory adhesion responses (DSP).^41^ Collectively, these age-associated changes highlight potential therapeutic vulnerabilities in older Lynch Syndrome patients. However, in the present study where age at diagnosis is known but age of the tumor is not established, we are unable to establish if the older patients that were diagnosed with more advanced tumors simply waited longer before presenting for care, and thus had more advanced tumors and a more differentiated proteome.

### Subtype-Specific Sex-Associated Differences

LS tumors showed notable sex-based proteomic divergence. Key metabolic and regulatory proteins – YME1L1, CAD, and PAPOLA – were differentially expressed between males and females. These suggest that male and female LS tumors may utilize distinct metabolic programs (mitochondrial function, nucleotide synthesis) and RNA processing mechanisms. Consistently, pathway analysis (Figure 7A) indicated enrichment of immune-related processes in one sex, including lymphopoiesis (lymphocyte development) and cytotoxic leukocyte activity, alongside predicted activation of the NF-κB transcription factor (RELA).^42^ This implies that the tumor immune microenvironment differs by sex in LS.

### Conclusions

To our knowledge, this is the first large-scale LC-MS/MS-based proteomics study that examines protein dysregulation across four colorectal cancer (CRC) subtypes—Lynch Syndrome (LS), Double Somatic (DS), MLH1 Hypermethylation (MLH1_hm_), and Microsatellite Stable (MSS)—while incorporating demographic richness and geographical specificity. By leveraging MS-based proteomics, we identified 1,084 proteins dysregulated across all subtypes, representing both common tumorigenic processes and subtype-specific regulatory mechanisms. Their differential expression patterns by age and sex highlight their potential as biomarkers for CRC classification, early detection, and patient stratification.

Our findings demonstrate that MSS tumors have a substantially different proteome compared to the three MSI-high cohorts. Also, the age of diagnosis significantly impacts LS and DS tumors, promoting tumor proliferation and metabolic shifts, while sex-based differences are most pronounced in LS and MLH1_hm_, driven by inflammation and immune regulation. This unique dataset provides a regionally specific and demographically rich resource, offering valuable insight into CRC cases.

This study fills a critical gap in LS proteomics research, where clinical samples have been historically limited. The identification of age- and sex-associated dysregulation patterns underscores the need for precision oncology approaches tailored to demographic factors, and the 1,084 shared proteins hold promise as non-invasive biomarkers for diagnostic and prognostic applications. The results support the development of demographic-aware therapeutic strategies and provide a framework for future studies on biomarker validation and clinical translation in CRC.

## METHODS

### Tissue Collection and Pulverization

Institutional review board approval was obtained from the Ohio State University (Study #2018C0146). CRC and normal tissues were obtained from the Biospecimen Services Shared Resource of the Ohio State University from participants who consented to the biorepository arm of the Ohio Colorectal Cancer Prevention Initiative (OCCPI).^14^ Methods for the OCCPI are described elsewhere, but briefly, 3310 patients who had surgical resection in Ohio for newly diagnosed colorectal adenocarcinoma were enrolled in the OCCPI between 2013 and 2016. A blood and two FFPE tissue samples (tumor and normal mucosa) were obtained from every participant. Fresh frozen tumor and normal tissue was obtained on a subset of patients who were diagnosed at Ohio State University. All patients received MMR deficiency screening and select patients underwent germline genetic testing (including all with MMR deficiency). From the 3310 patients enrolled, 142 were diagnosed with Lynch syndrome.^5, 14^

We conducted a label-free bottom-up proteomics analysis of 122 fresh frozen tumor and complimentary normal mucosa tissues from three types of MSI CRC samples: LS (n = 12), DS (n = 9), and *MLH1*_hm_ (n = 20), as well as MSS samples (n = 20), from a 61-patient cohort. (Figure 1 and Table 1). The entire sample set was blinded and randomized before any further sample preparation was conducted. Tissue chunks were immersed in liquid nitrogen and were pulverized into a fine powder using the Cellcrusher device (Cellcrusher, Schull, Cork, Ireland), which was pre-cooled in liquid nitrogen prior to use. The pulverized solid powder was then transferred to an ultracentrifuge tube, which was kept cooled in dry ice for the entire process. Twenty-four samples were partially un-blinded and grouped as A, B, C or D, which represented MSS, LS, DS or *MLH1*_hm_, respectively, and was designated as Batch 1. Batch 1 was used to generate a pooled sample for high pH Fractionation to create a spectral library. The entire sample set can be found in Table 1 and Figure 1.

### Tissue Lysis and Protein Digestion

Tissue lysis was conducted using a probe sonicator (Fisher Scientific) in pulse mode (1 second on, 2 seconds off) at 20% amplitude for a total of 1 minute sonication per sample. A bicinchoninic acid (BCA) assay was conducted using the Pierce BCA Protein Assay Kit (ThermoFisher Scientific, Rockford, Illinois, USA) to quantify the protein concentration. Afterward, 200 μg of protein for each sample was aliquoted on separate tubes and spun in a vacuum centrifuge to dryness. The protein digestion steps were conducted using the Suspension-Trapping method (ProtiFi, Fairpoint, New York, USA) with a few modifications.^43^ Briefly, each sample was resuspended in 50 μL lysis buffer containing 5% SDS and 5 mM triethylammonium bicarbonate (TEAB). DTT was added with a final concentration of 20 mM and samples were incubated in a thermomixer at 95 °C for 10 minutes. Iodoacetamide was added with a final concentration of 40 mM and samples were incubated at room temperature for 30 minutes in the dark, and then centrifuged for 8 minutes at 13,000 × g. Afterwards, 5 μL of 12% phosphoric acid was added to each sample and vortexed for a few seconds before adding 350 μL of a buffer solution containing (9:1) methanol and 1M TEAB. The resulting solutions were transferred individually to a corresponding well in the 96-well S-Trap digestion plate. The plate was subjected to a 1,500 g for 2 minutes in a centrifuge. The proteins were trapped in the filter of the plate and were washed with 200 μL of binding buffer, followed by centrifugation at 1,500 g for 2 minutes for each cycle. Afterward, the S-Trap digestion plate was placed on top of a clean receiver plate and 125 μL of digestion buffer, which contained 1:50 wt: wt a mix of Trypsin and Lys-C protease mix. The plate was incubated overnight at 37 °C in a humid environment. After the incubation, an additional 80 μL digestion buffer was added to all wells of the S-Trap digestion plate and was centrifuged at 1,500 g for 2 minutes. The eluted peptides in the collection plate were then subjected to vacuum centrifugation to dryness and stored at −20 °C until LC-MS/MS analysis.

### High-pH Fractionation of the Pooled Sample Group

To increase the protein sequence coverage of the study, protein digests from Batch 1 were subjected to an offline high-pH reversed-phase liquid-chromatography (RPLC) fractionation using the Pierce^™^ High pH Reversed-Phase Peptide Fractionation Kit (ThermoFisher Scientific, Rockford, Illinois, USA). A pooled digest was created by taking an equal volume from each sample in the Batch after resuspending in 0.1% formic acid. Following the manufacturer’s protocol, the sample was loaded into the fractionation spin column and eluted by different percentages of acetonitrile/water solution, resulting in 8 peptide fractions. This enabled the generation of a set of peptide fractions representing an equal representation of different patient cohorts analyzed by RPLC-MS/MS.

### RPLC-MS/MS Analysis

Prior to RPLC-MS/MS analysis, samples were resuspended with 0.1% formic acid solution. Protein identification was performed using nanoflow RPLC-MS/MS on a Bruker tims-TOF Pro equipped with a CaptiveSpray source operated in positive ion mode. Samples were separated on a C18 RPLC column (1.6 μm particle, 75 /360 μm i.d./o.d., 250 mm in length, IonOpticks) using a Bruker nanoElute UHPLC system. The column was equilibrated with 4 column volumes at 800 bar before each sample injection. Mobile phase A was 0.1% Formic Acid in water and acetonitrile (with 0.1% formic acid) was used as mobile phase B. A flow rate of 0.4 μL/min was used. Mobile phase B was increased from 2 to 17% over the first 40 minutes, then increased to 25% over the next 20 minutes, further increased to 37 % over the next 10 minutes, and finally increased to 80 % over 10 minutes and then held at 80 % for 10 minutes. MS and MS/MS experiments were recorded over the m/z range 100–1700 and 1/K0 of 0.6–1.6. Data-independent acquisition (DIA) and parallel accumulation-serial fragmentation (PASEF)^44, 45^ were employed for data acquisition. 32 mass steps per cycle were used over the mass range 400 to 1200 m/z and a mobility range of 0.61 to 1.49 1/K0 with a mass width of 25 Da with no mass or mobility overlap. Collision energy was increased with increasing 1/K0 and was set to 20 e1/K0 at 0.60 increasing to 59 at 1.60 1/K0.

Due to the high number of samples and chromatographic conditions of a 60-minute gradient program, we analyzed each sample once, resulting in a total of 122 total mass spectrometry runs.

### Spectral Library Generation using FragPipe and Identification and Quantification in DIA-NN

FragPipe (v. 18.0)^46^ was used to generate a spectral library from the DDA-PASEF runs of the offline fractionated samples of Batch 1. To align the retention times of the fractionated runs, including their technical replicate runs, the Common internal Retention Time standards (CiRT) alignment was turned on during the library generation. The target-decoy approach was used to filter the peptide and protein identifications.^47^ The library was filtered to 1% false discovery rate (FDR) at the protein and peptide levels. The spectral library contained 7744 proteins and was imported in DIA-NN.^48^ Bruker timsTOF.d files were directly imported into DIA-NN version 1.8 for peptide and protein identification and label-free quantification. Search parameters can be found in the Supplemental Information.

### Bioinformatics Analysis

#### Data Analysis in MSStats and Perseus

Using a DIA-based approach by diaPASEF and utilizing a spectral library generated from a pooled sample and further separated by offline – high pH fractionation, we initially identified 7,352 proteins in the study. We used the spectral library of the Using the R-based MSStats package, the DIA-NN output data matrix was analyzed with an accompanying “annotation” file, which included the condition, either as a “tumor” or “normal” tissue, and the specific CRC type.^49^ In turn, MSStats utilized the “paired” experimental design because each subject is represented by a normal and tumor biopsy sample. Statistically significant proteins (adjusted p-value of < 0.05) for each subtype were filtered and visualized their overlap using Venny 2.1,^50^ where we obtained the 1,084 proteins list which we considered the shared dysregulated proteins between the CRC subtypes. We further investigated the differential protein expressions of these proteins by using the Abundance Profiles feature in Persesus.^51^

#### GO/KEGG analysis

In the analysis conducted using the ‘clusterProfiler’ R package^52^, the ‘enrichKEGG’ function was employed with ‘hsa’ specified as the organism (representing Homo sapiens) and ‘org.Hs.eg.db’ selected as the organism database. To adjust p-values for controlling the FDR, the Benjamini-Hochberg (B.H.) procedure was utilized. The analysis set a p-value cutoff of 0.05 to identify statistically significant pathways, alongside a q-value cutoff of 0.2 to further mitigate the risk of false positives in the context of multiple hypothesis testing.

Similarly, the ‘enrichGO’ function from the same package was utilized, with ‘org.Hs.eg. db’ serving as the organism database. The B.H. method was again chosen for FDR adjustment. This function was applied to all three main Gene Ontology (G.O.) categories—Biological Process, Molecular Function, and Cellular Component—under the collective designation “ALL”. The criteria for statistical significance were maintained with a p-value cutoff of 0.05 and a q-value cutoff of 0.2, ensuring a consistent approach to controlling false discoveries across both enrichment analyses.

#### Graph analysis

To better understand the functional pathways underlying protein dysregulation in each CRC subtype, Gene Ontology (GO) enrichment analyses were performed on two tiers of protein sets: (1) significantly dysregulated proteins within each subtype, and (2) the top upregulated and downregulated proteins shared across all subtypes.

We next examined the statistical significance of sex-based differences in protein dysregulation across the CRC subtypes. P-values were calculated to evaluate how protein dysregulation levels differed between male and female patients, with lower p-values (-log_10_-transformed) indicating greater sex-associated differences. The analysis focused on the top proteins most significantly associated with sex-based dysregulation, visualized in the heatmap (Figure 6) and selected from a larger dataset, which included LS = 69, DS = 19, MLH1_hm_ = 63, and MSS = 10 proteins.

#### Analysis of Ribosomal Protein Expression

This analysis utilizes results from KEGG/GO analyses to identify proteins associated with ribosomes across various subtypes. A clustered heatmap is generated to visualize the abundance of these ribosomal-related proteins, employing an “Average” clustering method. This method calculates the distance between two clusters as the mean of all distances between their elements, enabling the identification of distinct expression patterns and similarities in ribosomal protein abundance across subtypes.

#### PCA Plot

For each subtype, the initial step involves identifying and matching each protein to its corresponding gene, while discarding proteins that lack gene matches. Subsequently, leveraging results from GO/KEGG analysis, the log2Fold Change (log2FC) data for each protein is compiled and cross-referenced with the Log (Intensity) information. The matched proteins are then utilized for Principal Component Analysis (PCA) plotting. The PCA plot creation process begins with standardizing the data, followed by reducing its dimensionality to two principal components. These components are visualized in a scatter plot, effectively illustrating the data’s distribution and variance within a two-dimensional space, providing a comprehensive view of the underlying biological processes.

#### Age-Related Expression Analysis

For each subtype, the first step was calculating a matrix of correlation coefficients between age and the expression levels of each protein by Pearson correlation. Proteins with correlation coefficients exceeding 0.5 or below −0.5 are selected for further visualization through heatmaps. These heatmaps display age-associated expression patterns, with a final merged heatmap integrating the filtered results from all subtypes to provide a comprehensive view of age-related expression dynamics.

#### Sex-Specific Expression Analysis

The analysis further segregates the dataset by sex to examine male and female expression profiles for each protein within the subtypes. For each sex, t-tests were utilized to determine the significance of observed differences, adopting a p-value threshold of 0.05. Proteins meeting this significance criterion are visualized in heatmaps, illustrating sex-specific expression patterns. A cumulative heatmap, merging results from all subtypes, offers an overarching perspective on sex influences in protein expression profiles.

We next investigated the correlation between patient age at diagnosis and protein dysregulation across the four CRC subtypes. For each subtype, a matrix of correlation coefficients was generated to assess the relationship between age and the dysregulation levels of each protein. Proteins with a correlation coefficient higher than 0.5 were considered positively correlated, indicating increased dysregulation in older patients, while those with a correlation coefficient lower than −0.5 were considered negatively correlated, suggesting decreased dysregulation with age at diagnosis. Figure 4 illustrates the top 20 age-correlated proteins, selected from a larger dataset, which includes DS = 196, LS = 271, MLH1_hm_ = 11, and MSS = 6 proteins. The larger dataset can be found in the Supplemental Information. The complete dataset was deposited to the ProteomeXchange Consortium^53^ via the PRIDE^54^ partner repository with the dataset identifier PXD073693.

## Supplementary Material

Supplement 1 **Table S1:** Top 10 Upregulated and Downregulated Proteins in Microsatellite Stable (MSS) Colorectal Cancer Subtype.
**Table S2:** Top 10 Upregulated and Downregulated Proteins in MLH1 Hypermethylation (MLH1_hm_) Colorectal Cancer Subtype
**Table S3:** Top 10 Upregulated and Downregulated Proteins in Double Somatic (DS) Colorectal Cancer Subtype
**Table S4:** Parameters used in the DIA-NN search.
**Table S5:** Significant proteins detected in each cohort. The data is available as a Excel file with separate tabs for each sample cohort and a tab with the 1084 dysregulated proteins.
**Figure S1:** Top Biological Process (BP) terms from Gene Ontology (GO) analysis for the Microsatellite Stable (MSS) subtype.
**Figure S2:** Top Biological Process (BP) terms from Gene Ontology (GO) analysis for the MLH1 Hypermethylation (MLH1hm) subtype.
**Figure S3:** Top Biological Process (BP) terms from Gene Ontology (GO) analysis for the Double Somatic (DS) subtype.
**Figure S4:** Subtype-specific age-associated protein expression trends in colorectal cancer.

Supplement 2

## Figures and Tables

**Table 1:** Characteristics of the CRC Cohort in this study.

CRC Subtype	Number of Patients, Sex	Range of Ages at Diagnosis (years)	Tumor Sites
Lynch Syndrome (LS)	n = 12 (75% female, 25% male)	25 – 57	Left = 4, Right = 7, Transverse = 2
Double Somatic (DS)	n = 9 (56 % female, 44% male)	27 – 80	Left = 2, Right = 7
MLH1 Hypermethylation (MLH1_hm_)	n = 20 (70% female, 30% male)	58 – 88	Left = 6, Right = 11, Transverse = 1
Microsatellite Stable (MSS)	n = 20 (40% female, 60% male)	51 – 90	Left = 11, Right = 4, Transverse = 2, NOS = 3
	**Total** = 61		

NOS = “Not otherwise specified.”

**Table 2:** Top 10 Upregulated and Downregulated Proteins in Lynch Syndrome (LS) Colorectal Cancer Subtype. The table displays the normalized Z-scores and log_2_ fold-changes (tumor tissue vs. normal mucosa) for the top 10 upregulated and downregulated proteins identified in the Lynch Syndrome (LS) subtype, based on the analysis of 1,084 common dysregulated proteins across colorectal cancer subtypes. The direction of regulation (upregulated or downregulated) is indicated for the LS subtype, and log_2_FC values and p-values from the other three subtypes (Double Somatic (DS), MLH1 Hypermethylation (MLH1_hm_), Microsatellite Stable (MSS)) are provided for comparison. The highlighted proteins correspond to the abundance trend plots in Figure 2D, which visualize global expression distributions and subtype-specific protein enrichment.

			*Microsatellite Stable (MSS)*		*MLH1 Hypermethylation (MLH1_hm_)*		*Lynch Syndrome (LS)*		*Double Somatic (DS)*	
*Protein*	Z-Score (LS)	Direction (LS)	log_2_FC	adj. p-value	log_2_FC	adj. p-value	log_2_FC	adj. p-value	log_2_FC	adj. p-value
*WDR82*	**1.50**	Up	0.54	1.04E-03	0.54	7.41E-04	0.77	4.90E-04	0.54	2.62E-02
*OGFR*	**1.49**	Up	1.12	1.64E-05	1.16	5.11E-06	1.45	2.13E-05	1.15	2.79E-03
*NOP16*	**1.49**	Up	1.02	1.71E-03	1.00	2.12E-03	1.19	6.32E-03	1.03	4.62E-02
*TGFB1*	**1.48**	Up	1.10	1.87E-03	1.06	1.33E-03	1.35	3.15E-03	1.11	3.15E-02
*YARS1*	**1.48**	Up	0.82	1.17E-04	0.83	5.64E-05	0.98	5.87E-04	0.85	8.13E-03
*RCN1*	**1.48**	Up	1.13	2.24E-06	1.09	1.65E-06	1.34	1.29E-05	1.08	1.78E-03
*PPID*	**1.47**	Up	0.70	6.52E-04	0.75	2.05E-04	1.02	3.69E-04	0.68	3.04E-02
*WBP11*	**1.47**	Up	0.93	1.28E-03	0.89	2.08E-03	1.09	4.43E-03	0.88	4.21E-02
*CNN2*	**1.45**	Up	0.97	9.22E-06	1.00	2.19E-06	1.26	1.18E-05	0.90	4.52E-03
*POLR2E*	**1.45**	Up	0.71	1.49E-03	0.74	8.22E-04	1.02	8.30E-04	0.80	1.85E-02
*F13A1*	**−1.46**	Down	−1.29	1.92E-04	−1.15	4.17E-04	−1.79	1.52E-04	−1.29	1.24E-02
*MTHFD1L*	**−1.47**	Down	1.79	6.38E-06	1.80	3.72E-06	1.42	3.86E-03	1.88	1.18E-03
*ZG16*	**−1.47**	Down	−2.63	7.42E-04	−2.44	9.37E-04	−3.33	1.47E-03	−2.47	4.50E-02
*RBM10*	**−1.47**	Down	0.84	1.25E-03	0.84	9.30E-04	0.74	3.02E-02	0.86	3.13E-02
*DKC1*	**−1.47**	Down	1.34	1.42E-04	1.32	9.65E-05	0.94	3.44E-02	1.26	1.63E-02
*GTF2F2*	**−1.49**	Down	1.23	8.18E-05	1.21	5.10E-05	0.99	1.19E-02	1.25	7.38E-03
*DIS3*	**−1.49**	Down	1.22	5.65E-07	1.21	2.75E-07	0.90	2.57E-03	1.17	1.09E-03
*ATP11C*	**−1.50**	Down	0.76	7.02E-05	0.77	3.69E-05	0.54	2.85E-02	0.78	5.92E-03
*DDX18*	**−1.50**	Down	1.20	1.42E-04	1.21	1.06E-04	1.12	6.01E-03	1.21	1.16E-02
*WDR43*	**−1.50**	Down	1.47	8.94E-06	1.49	2.88E-06	1.22	3.08E-03	1.49	2.34E-03

## References

[1] Organization WH. 2023. Available from: https://www.who.int/news-room/fact-sheets/detail/colorectal-cancer.

[2] Pearlman R, Haraldsdottir S, de la Chapelle A, Jonasson JG, Liyanarachchi S, Frankel WL, Rafnar T, Stefansson K, Pritchard CC, Hampel H. Clinical characteristics of patients with colorectal cancer with double somatic mismatch repair mutations compared with Lynch syndrome. J Med Genet. 2019;56(7):462–70. Epub 20190315. doi: 10.1136/jmedgenet-2018-105698. 30877237 PMC6748629

[3] Rigter LS, Snaebjornsson P, Rosenberg EH, Atmodimedjo PN, Aleman BM, Ten Hoeve J, Geurts-Giele WR, group P, van Ravesteyn TW, Hoeksel J, Meijer GA, Te Riele H, van Leeuwen FE, Dinjens WN, van Leerdam ME. Double somatic mutations in mismatch repair genes are frequent in colorectal cancer after Hodgkin’s lymphoma treatment. Gut. 2018;67(3):447–55. doi: 10.1136/gutjnl-2016-312608. 29439113

[4] Toyota M, Ahuja N, Ohe-Toyota M, Herman JG, Baylin SB, Issa JP. CpG island methylator phenotype in colorectal cancer. Proc Natl Acad Sci U S A. 1999;96(15):8681–6. doi: 10.1073/pnas.96.15.8681. 10411935 PMC17576

[5] Pearlman R, Frankel WL, Swanson B, Zhao W, Yilmaz A, Miller K, Bacher J, Bigley C, Nelsen L, Goodfellow PJ, Goldberg RM, Paskett E, Shields PG, Freudenheim JL, Stanich PP, Lattimer I, Arnold M, Liyanarachchi S, Kalady M, Heald B, Greenwood C, Paquette I, Prues M, Draper DJ, Lindeman C, Kuebler JP, Reynolds K, Brell JM, Shaper AA, Mahesh S, Buie N, Weeman K, Shine K, Haut M, Edwards J, Bastola S, Wickham K, Khanduja KS, Zacks R, Pritchard CC, Shirts BH, Jacobson A, Allen B, de la Chapelle A, Hampel H, Ohio Colorectal Cancer Prevention Initiative Study G. Prevalence and Spectrum of Germline Cancer Susceptibility Gene Mutations Among Patients With Early-Onset Colorectal Cancer. JAMA Oncol. 2017;3(4):464–71. doi: 10.1001/jamaoncol.2016.5194. 27978560 PMC5564179

[6] Nusinow DP, Szpyt J, Ghandi M, Rose CM, McDonald ER 3rd, Kalocsay M, Jane-Valbuena J, Gelfand E, Schweppe DK, Jedrychowski M, Golji J, Porter DA, Rejtar T, Wang YK, Kryukov GV, Stegmeier F, Erickson BK, Garraway LA, Sellers WR, Gygi SP. Quantitative Proteomics of the Cancer Cell Line Encyclopedia. Cell. 2020;180(2):387–402 e16. doi: 10.1016/j.cell.2019.12.023. 31978347 PMC7339254

[7] Goncalves E, Poulos RC, Cai Z, Barthorpe S, Manda SS, Lucas N, Beck A, Bucio-Noble D, Dausmann M, Hall C, Hecker M, Koh J, Lightfoot H, Mahboob S, Mali I, Morris J, Richardson L, Seneviratne AJ, Shepherd R, Sykes E, Thomas F, Valentini S, Williams SG, Wu Y, Xavier D, MacKenzie KL, Hains PG, Tully B, Robinson PJ, Zhong Q, Garnett MJ, Reddel RR. Pan-cancer proteomic map of 949 human cell lines. Cancer Cell. 2022;40(8):835–49 e8. Epub 20220714. doi: 10.1016/j.ccell.2022.06.010. 35839778 PMC9387775

[8] Cancer Genome Atlas N. Comprehensive molecular characterization of human colon and rectal cancer. Nature. 2012;487(7407):330–7. Epub 20120718. doi: 10.1038/nature11252. 22810696 PMC3401966

[9] Zhang B, Wang J, Wang X, Zhu J, Liu Q, Shi Z, Chambers MC, Zimmerman LJ, Shaddox KF, Kim S, Davies SR, Wang S, Wang P, Kinsinger CR, Rivers RC, Rodriguez H, Townsend RR, Ellis MJ, Carr SA, Tabb DL, Coffey RJ, Slebos RJ, Liebler DC, Nci C. Proteogenomic characterization of human colon and rectal cancer. Nature. 2014;513(7518):382–7. Epub 20140720. doi: 10.1038/nature13438. 25043054 PMC4249766

[10] Tanaka A, Ogawa M, Zhou Y, Namba K, Hendrickson RC, Miele MM, Li Z, Klimstra DS, Buckley PG, Gulcher J, Wang JY, Roehrl MHA. Proteogenomic characterization of primary colorectal cancer and metastatic progression identifies proteome-based subtypes and signatures. Cell Rep. 2024;43(2):113810. Epub 20240219. doi: 10.1016/j.celrep.2024.113810. 38377004 PMC11288375

[11] Jiang Y, Sun A, Zhao Y, Ying W, Sun H, Yang X, Xing B, Sun W, Ren L, Hu B, Li C, Zhang L, Qin G, Zhang M, Chen N, Zhang M, Huang Y, Zhou J, Zhao Y, Liu M, Zhu X, Qiu Y, Sun Y, Huang C, Yan M, Wang M, Liu W, Tian F, Xu H, Zhou J, Wu Z, Shi T, Zhu W, Qin J, Xie L, Fan J, Qian X, He F, Chinese Human Proteome Project C. Proteomics identifies new therapeutic targets of early-stage hepatocellular carcinoma. Nature. 2019;567(7747):257–61. Epub 20190227. doi: 10.1038/s41586-019-0987-8. 30814741

[12] Fang F, Fries B, Wang Z, Liu X, Hummon AB, Sun L. Quantitative Top-Down Proteomics Reveals Significant Differences in Histone Proteoforms Between Metastatic and Nonmetastatic Colorectal Cancer Cells. Proteomics. 2025;25(24):88–98. Epub 20250417. doi: 10.1002/pmic.202400336. 40243858 PMC12716108

[13] McCool EN, Xu T, Chen W, Beller NC, Nolan SM, Hummon AB, Liu X, Sun L. Deep top-down proteomics revealed significant proteoform-level differences between metastatic and nonmetastatic colorectal cancer cells. Sci Adv. 2022;8(51):eabq6348. Epub 20221221. doi: 10.1126/sciadv.abq6348. 36542699 PMC9770947

[14] Pearlman R, Frankel WL, Swanson BJ, Jones D, Zhao W, Yilmaz A, Miller K, Bacher J, Bigley C, Nelsen L, Goodfellow PJ, Goldberg RM, Paskett E, Shields PG, Freudenheim JL, Stanich PP, Lattimer I, Arnold M, Prior TW, Haut M, Kalady MF, Heald B, Paquette I, Draper DJ, Brell JM, Mahesh S, Weeman K, Bastola S, Zangmeister J, Gowda A, Kencana F, Malcolm A, Liu Y, Cole S, Bane C, Li C, Rehmus E, Pritchard CC, Shirts BH, Jacobson A, Cummings SA, de la Chapelle A, Hampel H. Prospective Statewide Study of Universal Screening for Hereditary Colorectal Cancer: The Ohio Colorectal Cancer Prevention Initiative. JCO Precis Oncol. 2021;5. Epub 20210505. doi: 10.1200/PO.20.00525. PMC8232834 34250417

[15] Wodzinski D, Wosiak A, Pietrzak J, Swiechowski R, Kordek R, Balcerczak E. Assessment of the TGFB1 gene expression and methylation status of the promoter region in patients with colorectal cancer. Sci Rep. 2022;12(1):11488. Epub 20220707. doi: 10.1038/s41598-022-15599-4. 35798776 PMC9263105

[16] Liu Y, Yang J, Han W, Gu T, Yao L, Wang Y, Chen H. Identification and validation of metastasis-related gene ZG16 in the prognosis and progression in colorectal cancer. Front Oncol. 2024;14:1409329. Epub 20240724. doi: 10.3389/fonc.2024.1409329. 39114307 PMC11303331

[17] Meng H, Ding Y, Liu E, Li W, Wang L. ZG16 regulates PD-L1 expression and promotes local immunity in colon cancer. Transl Oncol. 2021;14(2):101003. Epub 20201225. doi: 10.1016/j.tranon.2020.101003. 33360840 PMC7773682

[18] Nait Slimane S, Marcel V, Fenouil T, Catez F, Saurin JC, Bouvet P, Diaz JJ, Mertani HC. Ribosome Biogenesis Alterations in Colorectal Cancer. Cells. 2020;9(11). Epub 20201027. doi: 10.3390/cells9112361. PMC7693311 33120992

[19] Bai XF, Liu JW. Identification of RNA-Binding Proteins with Prognostic Prediction in Colorectal Cancer. Biomed Res Int. 2021;2021:7114445. Epub 20211105. doi: 10.1155/2021/7114445. 34778456 PMC8589521

[20] de The H. Differentiation therapy revisited. Nat Rev Cancer. 2018;18(2):117–27. Epub 20171201. doi: 10.1038/nrc.2017.103. 29192213

[21] Nguyen LH, Guo CG, Ma W, Giovannucci EL, Strate LL, Chan AT. Recent, Mid, and Late Adulthood Antibiotic Use Are Associated With Subsequent Risk of Diverticulitis. Gastroenterology. 2021;160(6):2172–4 e3. Epub 20210120. doi: 10.1053/j.gastro.2021.01.200. 33484687 PMC8459316

[22] Martin-Garcia D, Garcia-Aranda M, Redondo M. Biomarker Identification through Proteomics in Colorectal Cancer. Int J Mol Sci. 2024;25(4). Epub 20240214. doi: 10.3390/ijms25042283. PMC10888664 38396959

[23] Li Y, Wang B, Ma F, Jiang D, Wang Y, Li K, Tan S, Feng J, Wang Y, Qin Z, Xu G, Tian S, Zhang X, Xu C, Wu J, Xu J, Hou Y, Ding C. Proteomic characterization of the colorectal cancer response to chemoradiation and targeted therapies reveals potential therapeutic strategies. Cell Rep Med. 2023;4(12):101311. Epub 20231211. doi: 10.1016/j.xcrm.2023.101311. 38086380 PMC10772406

[24] Hao JJ, Zhi X, Wang Y, Zhang Z, Hao Z, Ye R, Tang Z, Qian F, Wang Q, Zhu J. Comprehensive Proteomic Characterization of the Human Colorectal Carcinoma Reveals Signature Proteins and Perturbed Pathways. Sci Rep. 2017;7:42436. Epub 20170209. doi: 10.1038/srep42436. 28181595 PMC5299448

[25] Sonenberg N, Hinnebusch AG. Regulation of translation initiation in eukaryotes: mechanisms and biological targets. Cell. 2009;136(4):731–45. doi: 10.1016/j.cell.2009.01.042. 19239892 PMC3610329

[26] Polunovsky VA, Rosenwald IB, Tan AT, White J, Chiang L, Sonenberg N, Bitterman PB. Translational control of programmed cell death: eukaryotic translation initiation factor 4E blocks apoptosis in growth-factor-restricted fibroblasts with physiologically expressed or deregulated Myc. Mol Cell Biol. 1996;16(11):6573–81. doi: 10.1128/MCB.16.11.6573. 8887686 PMC231659

[27] Holland EC, Sonenberg N, Pandolfi PP, Thomas G. Signaling control of mRNA translation in cancer pathogenesis. Oncogene. 2004;23(18):3138–44. doi: 10.1038/sj.onc.1207590. 15094763

[28] Alboushi L, Hackett AP, Naeli P, Bakhti M, Jafarnejad SM. Multifaceted control of mRNA translation machinery in cancer. Cell Signal. 2021;84:110037. Epub 20210508. doi: 10.1016/j.cellsig.2021.110037. 33975011

[29] Meng H, Li W, Boardman LA, Wang L. Loss of ZG16 is associated with molecular and clinicopathological phenotypes of colorectal cancer. BMC Cancer. 2018;18(1):433. Epub 20180416. doi: 10.1186/s12885-018-4337-2. 29661177 PMC5902988

[30] Chen X, Chen Y, Zhang Y, Zhang Y, Wang Y, Li Y, Sun Y, Meng G, Yang G, Li H. ZG16 impacts gut microbiota-associated intestinal inflammation and pulmonary mucosal function through bacterial metabolites. Int Immunopharmacol. 2024;141:112995. Epub 20240827. doi: 10.1016/j.intimp.2024.112995. 39191121

[31] Lindhorst PH, Hummon AB. Proteomics of Colorectal Cancer: Tumors, Organoids, and Cell Cultures-A Minireview. Front Mol Biosci. 2020;7:604492. Epub 20201210. doi: 10.3389/fmolb.2020.604492. 33363210 PMC7758474

[32] Fumarola S, Cianfruglia L, Cecati M, Giammarchi C, Vaiasicca S, Gasparrini M. Polyphenol Intake in Elderly Patients: A Novel Approach to Counteract Colorectal Cancer Risk? Int J Mol Sci. 2025;26(6). Epub 20250311. doi: 10.3390/ijms26062497. 40141143 PMC11942013

[33] Itatani Y, Kawada K, Sakai Y. Treatment of Elderly Patients with Colorectal Cancer. Biomed Res Int. 2018;2018:2176056. Epub 20180311. doi: 10.1155/2018/2176056. 29713641 PMC5866880

[34] Huang ZY, Zhang LH, Zhao C, Liu R, Tong H, Gan C, Lan T, Tang CW, Gao JH. High HIF-1alpha expression predicts poor prognosis of patients with colon adenocarcinoma. Int J Clin Exp Pathol. 2018;11(12):5635–46. Epub 20181201. 31949650 PMC6963075

[35] Gong Y, Liu Y, Wang T, Li Z, Gao L, Chen H, Shu Y, Li Y, Xu H, Zhou Z, Dai L. Age-Associated Proteomic Signatures and Potential Clinically Actionable Targets of Colorectal Cancer. Mol Cell Proteomics. 2021;20:100115. Epub 20210612. doi: 10.1016/j.mcpro.2021.100115. 34129943 PMC8441843

[36] Cai X, Liu C, Zhang TN, Zhu YW, Dong X, Xue P. Down-regulation of FN1 inhibits colorectal carcinogenesis by suppressing proliferation, migration, and invasion. J Cell Biochem. 2018;119(6):4717–28. Epub 20180228. doi: 10.1002/jcb.26651. 29274284

[37] Young J, Simms LA, Biden KG, Wynter C, Whitehall V, Karamatic R, George J, Goldblatt J, Walpole I, Robin SA, Borten MM, Stitz R, Searle J, McKeone D, Fraser L, Purdie DR, Podger K, Price R, Buttenshaw R, Walsh MD, Barker M, Leggett BA, Jass JR. Features of colorectal cancers with high-level microsatellite instability occurring in familial and sporadic settings: parallel pathways of tumorigenesis. Am J Pathol. 2001;159(6):2107–16. doi: 10.1016/S0002-9440(10)63062-3. 11733361 PMC1850604

[38] Byrne JA, Frost S, Chen Y, Bright RK. Tumor protein D52 (TPD52) and cancer-oncogene understudy or understudied oncogene? Tumour Biol. 2014;35(8):7369–82. Epub 20140506. doi: 10.1007/s13277-014-2006-x. 24798974

[39] Ummanni R, Teller S, Junker H, Zimmermann U, Venz S, Scharf C, Giebel J, Walther R. Altered expression of tumor protein D52 regulates apoptosis and migration of prostate cancer cells. FEBS J. 2008;275(22):5703–13. doi: 10.1111/j.1742-4658.2008.06697.x. 18959755

[40] Moriwaki K, Miyoshi E. Fucosylation and gastrointestinal cancer. World J Hepatol. 2010;2(4):151–61. doi: 10.4254/wjh.v2.i4.151. 21160988 PMC2999278

[41] Yang L, Chen Y, Cui T, Knosel T, Zhang Q, Albring KF, Huber O, Petersen I. Desmoplakin acts as a tumor suppressor by inhibition of the Wnt/beta-catenin signaling pathway in human lung cancer. Carcinogenesis. 2012;33(10):1863–70. Epub 20120712. doi: 10.1093/carcin/bgs226. 22791817

[42] Rodriguez-Santiago Y, Garay-Canales CA, Nava-Castro KE, Morales-Montor J. Sexual dimorphism in colorectal cancer: molecular mechanisms and treatment strategies. Biol Sex Differ. 2024;15(1):48. Epub 20240612. doi: 10.1186/s13293-024-00623-1. 38867310 PMC11170921

[43] Zougman A, Selby PJ, Banks RE. Suspension trapping (STrap) sample preparation method for bottom-up proteomics analysis. Proteomics. 2014;14(9):1006–0. Epub 20140326. doi: 10.1002/pmic.201300553. 24678027

[44] Ludwig C, Gillet L, Rosenberger G, Amon S, Collins BC, Aebersold R. Data-independent acquisition-based SWATH-MS for quantitative proteomics: a tutorial. Mol Syst Biol. 2018;14(8):e8126. Epub 20180813. doi: 10.15252/msb.20178126. 30104418 PMC6088389

[45] Meier F, Beck S, Grassl N, Lubeck M, Park MA, Raether O, Mann M. Parallel Accumulation-Serial Fragmentation (PASEF): Multiplying Sequencing Speed and Sensitivity by Synchronized Scans in a Trapped Ion Mobility Device. J Proteome Res. 2015;14(12):5378–87. Epub 20151113. doi: 10.1021/acs.jproteome.5b00932. 26538118

[46] Kong AT, Leprevost FV, Avtonomov DM, Mellacheruvu D, Nesvizhskii AI. MSFragger: ultrafast and comprehensive peptide identification in mass spectrometry-based proteomics. Nat Methods. 2017;14(5):513–20. Epub 20170410. doi: 10.1038/nmeth.4256. 28394336 PMC5409104

[47] Elias JE, Gygi SP. Target-decoy search strategy for increased confidence in large-scale protein identifications by mass spectrometry. Nat Methods. 2007;4(3):207–14. doi: 10.1038/nmeth1019. 17327847

[48] Demichev V, Messner CB, Vernardis SI, Lilley KS, Ralser M. DIA-NN: neural networks and interference correction enable deep proteome coverage in high throughput. Nat Methods. 2020;17(1):41–4. Epub 20191125. doi: 10.1038/s41592-019-0638-x. 31768060 PMC6949130

[49] Kohler D, Staniak M, Tsai TH, Huang T, Shulman N, Bernhardt OM, MacLean BX, Nesvizhskii AI, Reiter L, Sabido E, Choi M, Vitek O. MSstats Version 4.0: Statistical Analyses of Quantitative Mass Spectrometry-Based Proteomic Experiments with Chromatography-Based Quantification at Scale. J Proteome Res. 2023;22(5):1466–82. Epub 20230405. doi: 10.1021/acs.jproteome.2c00834. 37018319 PMC10629259

[50] Oliveros JC. Venny 2.1. Available from: https://bioinfogp.cnb.csic.es/tools/venny/.

[51] Tyanova S, Temu T, Sinitcyn P, Carlson A, Hein MY, Geiger T, Mann M, Cox J. The Perseus computational platform for comprehensive analysis of (prote)omics data. Nat Methods. 2016;13(9):731–40. Epub 20160627. doi: 10.1038/nmeth.3901. 27348712

[52] Yu G, Wang LG, Han Y, He QY. clusterProfiler: an R package for comparing biological themes among gene clusters. OMICS. 2012;16(5):284–7. Epub 20120328. doi: 10.1089/omi.2011.0118. 22455463 PMC3339379

[53] Deutsch EW, Bandeira N, Perez-Riverol Y, Sharma V, Carver JJ, Mendoza L, Kundu DJ, Bandla C, Kamatchinathan S, Hewapathirana S, Sun Z, Kawano S, Okuda S, Connolly B, MacLean B, MacCoss MJ, Chen T, Zhu Y, Ishihama Y, Vizcaino JA. The ProteomeXchange consortium in 2026: making proteomics data FAIR. Nucleic Acids Res. 2026;54(D1):D459–D69. doi: 10.1093/nar/gkaf1146. 41206473 PMC12807779

[54] Perez-Riverol Y, Bandla C, Kundu DJ, Kamatchinathan S, Bai J, Hewapathirana S, John NS, Prakash A, Walzer M, Wang S, Vizcaino JA. The PRIDE database at 20 years: 2025 update. Nucleic Acids Res. 2025;53(D1):D543–D53. doi: 10.1093/nar/gkae1011. 39494541 PMC11701690

